# Pleural aspergillosis complicated by recurrent pneumothorax: a case report

**DOI:** 10.1186/1752-1947-4-180

**Published:** 2010-06-17

**Authors:** Weihua Zhang, Ye Hu, Liang'an Chen, Jie Gao, Lixin Xie

**Affiliations:** 1 Department of Respiratory Medicine, Chinese PLA General Hospital, 28th Fuxing Road, Beijing, 100853, China; 2 Department of Pathology, Chinese PLA General Hospital, Beijing, 100853, China

## Abstract

**Introduction:**

Pneumothorax as the first symptom of pleural aspergillosis is rare.

**Case presentation:**

A 31-year-old asthmatic Chinese man presented with recurrent spontaneous pneumothorax and underwent lobectomy due to persistent air leakage. Aspergillus was detected histopathologically in the visceral pleural cavity. He was treated with itraconazole at 200 mg a day, and nine months later he had no recurrent pneumothorax or aspergillus infection.

**Conclusion:**

Recurrent pneumothorax may be a rare manifestation of aspergillus infection. Aspergillus species infection should be considered in the differential diagnosis of recurrent pneumothorax patients, particularly those with chronic lung disease.

## Introduction

Aspergillus spp. are ubiquitous fungi that are acquired by inhalation of airborne spores and may cause a variety of clinical lung syndromes. The severity of lung aspergillosis depends upon immune status and the presence of underlying lung disease. The manifestations range from invasive pulmonary aspergillosis in severely immunocompromised patients to chronic necrotizing aspergillosis in patients with chronic lung disease and/or mildly compromised immune systems. Aspergilloma is primarily seen in patients with cavitary lung disease, while acute bronchopulmonary aspergillosis (ABPA) is a hypersensitivity disease of the lungs that almost always affects patients with asthma or cystic fibrosis. In this paper, we report a rare case of a patient who had multiple blebs, recurrent pneumothorax and pleura that was infected by aspergillus, with clinical features that were different from those described in the literature [1].

## Case presentation

**Figure 1:**
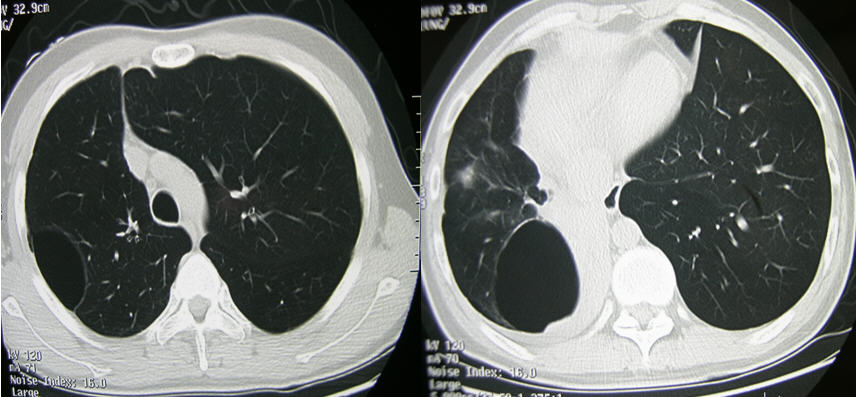
**Chest CT shows multiple blebs in right upper lobe and middle lobe, right lower lobe collapse**.

A 31-year-old Chinese man was referred for recurrent pneumothorax and right lower lobe atelectasis with two occurrences of spontaneous pneumothorax during the previous four months. He had a 20-year history of asthmatic disease and no smoking history. He had irregularly taken 5 to 10 mg of oral prednisone per day for half a year. On admission, chest computed tomography (CT) showed multiple lung blebs in the upper lobes, a large bleb in the right middle lobe, and right lower lobe collapse (Figure 1).

Laboratory tests showed that total IgG, IgM and IgA levels were normal, and that IgE was 263 IU/mL (normal level: 0-100 IU/mL). He had 7% eosinophils. Erythrocyte sedimentation rate was 30 mm/h. Flexible fiberoptic bronchoscopy showed mucosal hyperemia and purulent secretions in the right lower lobe. Cytological classification examination by bronchoscopy showed that bronchial alveolar lavage fluid (BALF) was normal. Bacterial and fungal smears and cultures of BALF were negative. He was discharged after these evaluations.

**Figure 2:**
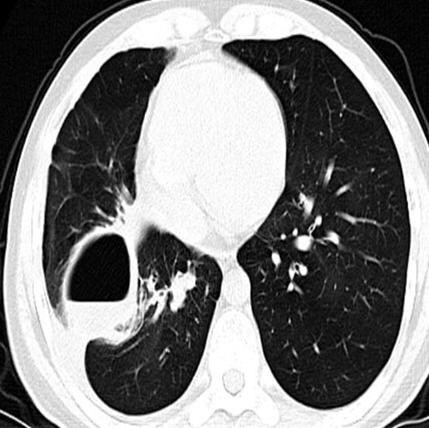
**Chest CT shows the right lower lobe re-expansion, the bleb in right middle lobe aggravated into a large cyst with thick wall and fluid-level**.

**Figure 3:**
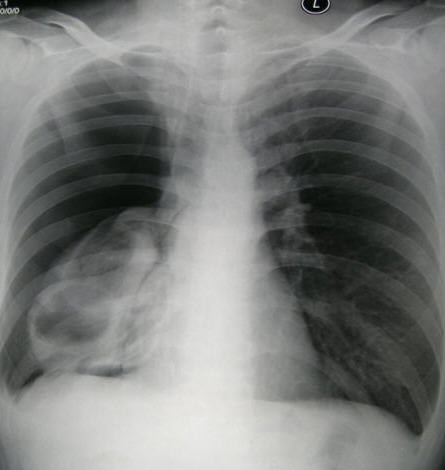
**Chest X-ray showed a right pneumothorax**.

Two weeks later he was hospitalized again because of a low-grade fever. Follow-up chest CT scan showed that the right lower lobe had re-expanded and that the bleb in the middle lobe had become aggravated into a cyst with a thick wall and fluid-level (Figure 2). After this admission, right spontaneous pneumothorax occurred again (Figure 3). Because of persistent air leakage, a right middle lobectomy and loop ligature of the blebs in the upper and lower lobes of the right lung were performed.

**Figure 4:**
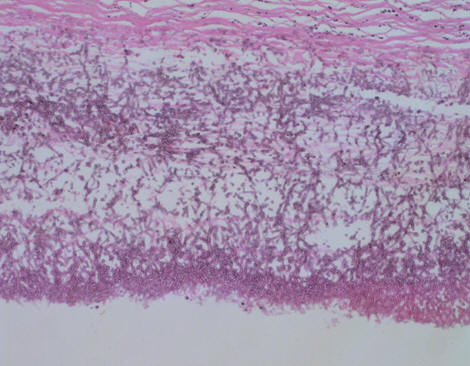
**In the resected specimen, Y-shape black aspergillus mycelia with septa were recognized (hematoxylin and eosin × 100)**.

**Figure 5:**
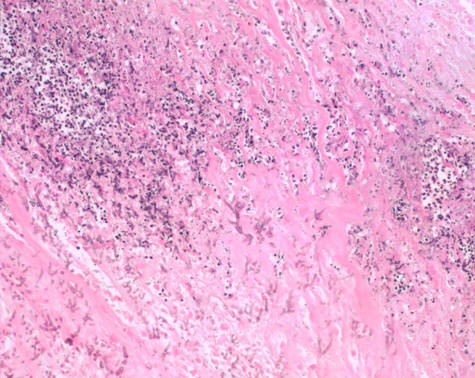
Y-shape black aspergillus mycelia with septa and inflammatory cells were also seen in hypertrophic visceral pleura (hematoxylin and eosin × 100)

**Figure 6:**
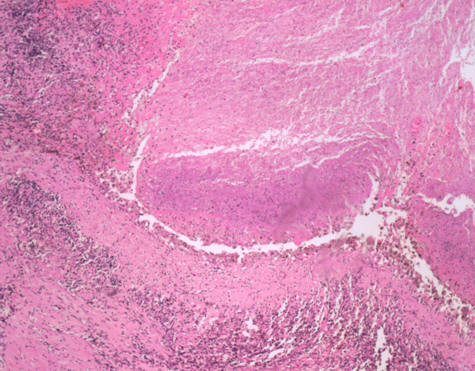
**Necrosis of lung tissue and alveoli hemorrhage (hematoxylin and eosin × 40)**.

Aspergillus hyphae were found in the hypertrophic visceral pleural and pleural cavity. Chronic inflammation, necrosis and alveoli hemorrhage were also seen in the resected right middle lobe (Figures 4, 5, 6). Serum tests for *Aspergillus galactomannan *antigen and beta D-glucan were negative. Based on the histological results, the patient met the criteria for a diagnosis of fungal lung disease. He was treated with itraconazole at 200 mg a day, and nine months later he no longer had pneumothorax.

## Discussion

Pneumothorax as the first symptom of aspergillosis is rare. To date, only a few cases have been reported in a search of PubMed. Of all reported cases, two had lung abscesses [2-9]. Any causal connection between pneumothorax and aspergillus infection was not clear. We believe that pneumothorax in our case was caused by rupture of a subpleural bleb. The three occurrences of spontaneous pneumothorax in less than half a year indicated that some underlying cause resulted in progressive hyperinflation and damaged these blebs.

Aspergillus spp. are ubiquitous fungi that are acquired by inhalation of airborne spores. Aspergillus airway colonization usually occurs in patients with an underlying chronic airway disease, such as asthma, bronchiectasis or cystic fibrosis. The airway sequelae of chronic airway disease, such as cellular debris, increased mucus, cavities and ectatic bronchi, are important for trapping aspergillus spores. It has been reported that aspergillus colonization may occur in more than 25% of asthmatic patients [10].

In our case, pulmonary sequelae due to asthma and long-term oral corticosteroid use favored airway colonization and invasion by aspergillus. No other micro-organisms, except for mycelia with septae, were found in the resected biopsy. We assumed that aspergillus colonization and the amount of hyphae had caused partial obstruction of small bronchioles. Partial obstruction of small bronchioles may have acted as a check-valve, which caused blebs and the subsequent progressive hyperinflation. It was interesting that at two or three weeks after a bronchoscopy investigation and bronchial alveolar lavage, the patient developed a low-grade fever. A CT scan on the second admission showed that the original bleb in the right middle lobe had developed a cyst with a thick wall and fluid level. Hence, the bronchoscopy investigation might have aggravated the aspergillus infection [11].

Pleural aspergillosis is an uncommon disease. It mostly occurs in patients with an established empyema and a bronchopleural fistula or pleurocutaneous fistula. The diagnosis of pleural aspergillosis in our case was established by demonstrating the organism in a resected specimen. Biopsy specimens showed micronodular mycetomas with septate hyphae that were highly suggestive of aspergillus. Unlike other reported cases, our patient had no bronchopleural or pleurocutaneous fistula, so we believed that the only possible route by which aspergillus had reached the pleura was through pneumothorax.

There is no consensus on the treatment for such rare cases. Our patient had undergone an emergency lung resection due to pneumothorax and, afterward, had taken itraconazole at 200 mg a day. Nine months later he had no recurrent pneumothorax or aspergillus infection. Surgical resection of infected lung tissue combined with a long-term anti-fungal agent may improve the prognosis for patients with this condition.

## Conclusions

In conclusion, recurrent pneumothorax and pleural aspergillosis may be rare manifestations of aspergillus infection. Aspergillus spp. infection should be considered in the differential diagnosis of such cases, particularly for those with a chronic lung disease.

## Consent

Written informed consent was obtained from the patient for publication of this case report and any accompanying images. A copy of the written consent is available for review by the Editor-in-Chief of this journal.

## Competing interests

The authors declare that they have no competing interests.

## Authors' contributions

ZWH, HY and XLX were involved with patient management and writing the manuscript. GJ performed the histological examination of the biopsy. CHA was involved with patient management. All authors read and approved the final manuscript.
